# Supplementary material for: Place fields of single spikes in hippocampus involve Kcnq3 channel-dependent entrainment of complex spike bursts
Source: Nat Commun. 2021 Aug 10;12:4801. doi: 10.1038/s41467-021-24805-2 (PMC8355348; doi:10.1038/s41467-021-24805-2)
Supplement: Supplementary file 1 — Supplementary Information [file 41467_2021_24805_MOESM1_ESM.pdf]

Supplementary Information

Supplementary Figures

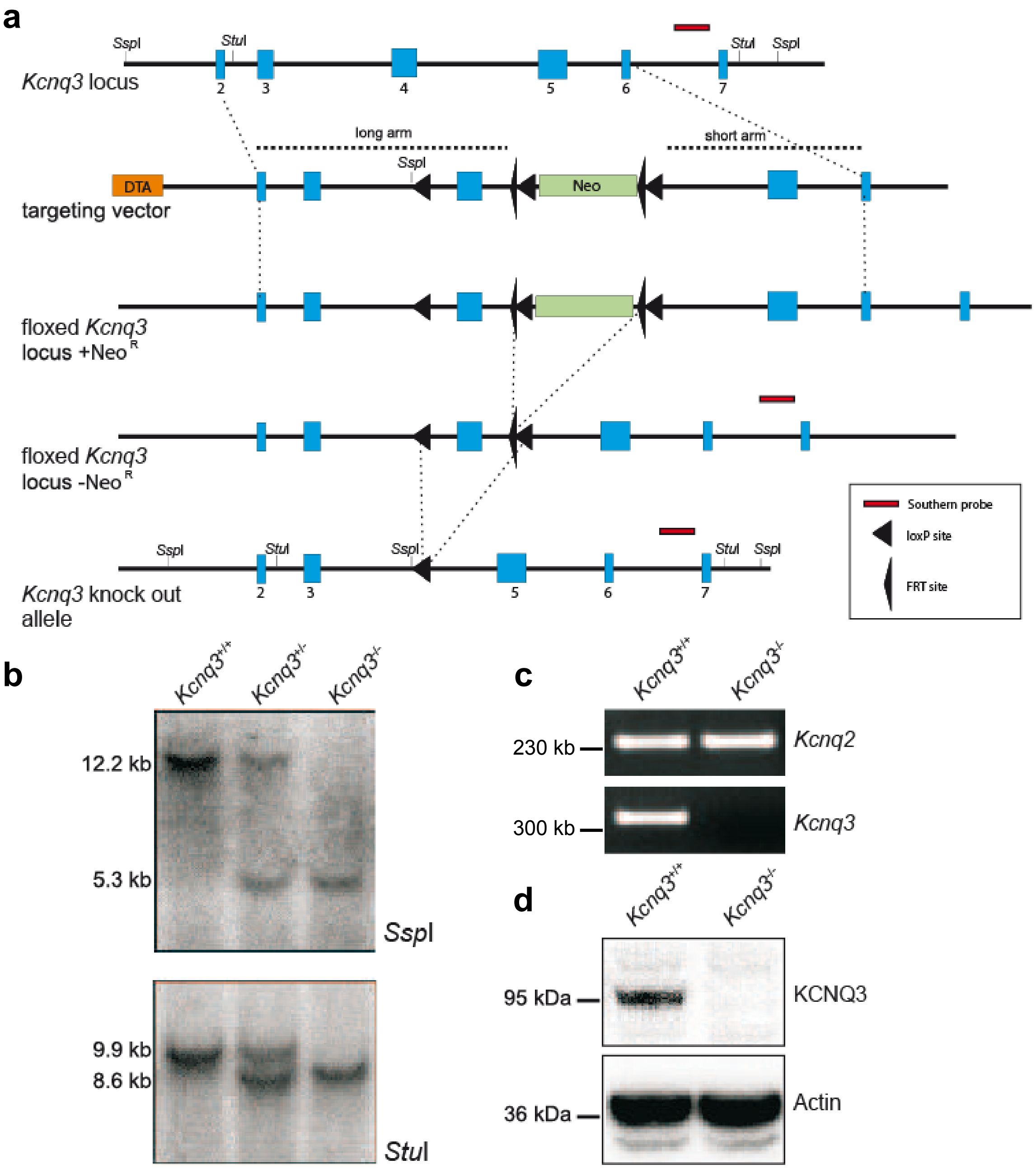

Supplementary Figure 1. Generation of *Kcnq3*$^{-/-}$ mice.

a *Kcnq3* genomic locus that encompasses exon 2 through 7 and targeting vector. The *Neo*^{R} cassette was removed by FLPe-recombination in mice resulting in exon 4 flanked by loxP sites. Cre-mediated recombination results in excision of exon 4. **b** Southern blot analysis using *Ssp*I (top) and *Stu*I (bottom) digested DNA from tail biopsies of *Kcnq3*$^{+/+}$, *Kcnq3*$^{+/-}$ and *Kcnq3*$^{-/-}$ mice confirms successful deletion of exon 4. **c** RT-PCR analysis of total brain RNA from *Kcnq3*$^{+/+}$and *Kcnq3*$^{-/-}$ mice confirms absence of *Kcnq3*$^{-/-}$mRNA. **d** Loss of KCNQ3 protein in *Kcnq3*$^{-/-}$ brain confirmed by Western Blot analysis.

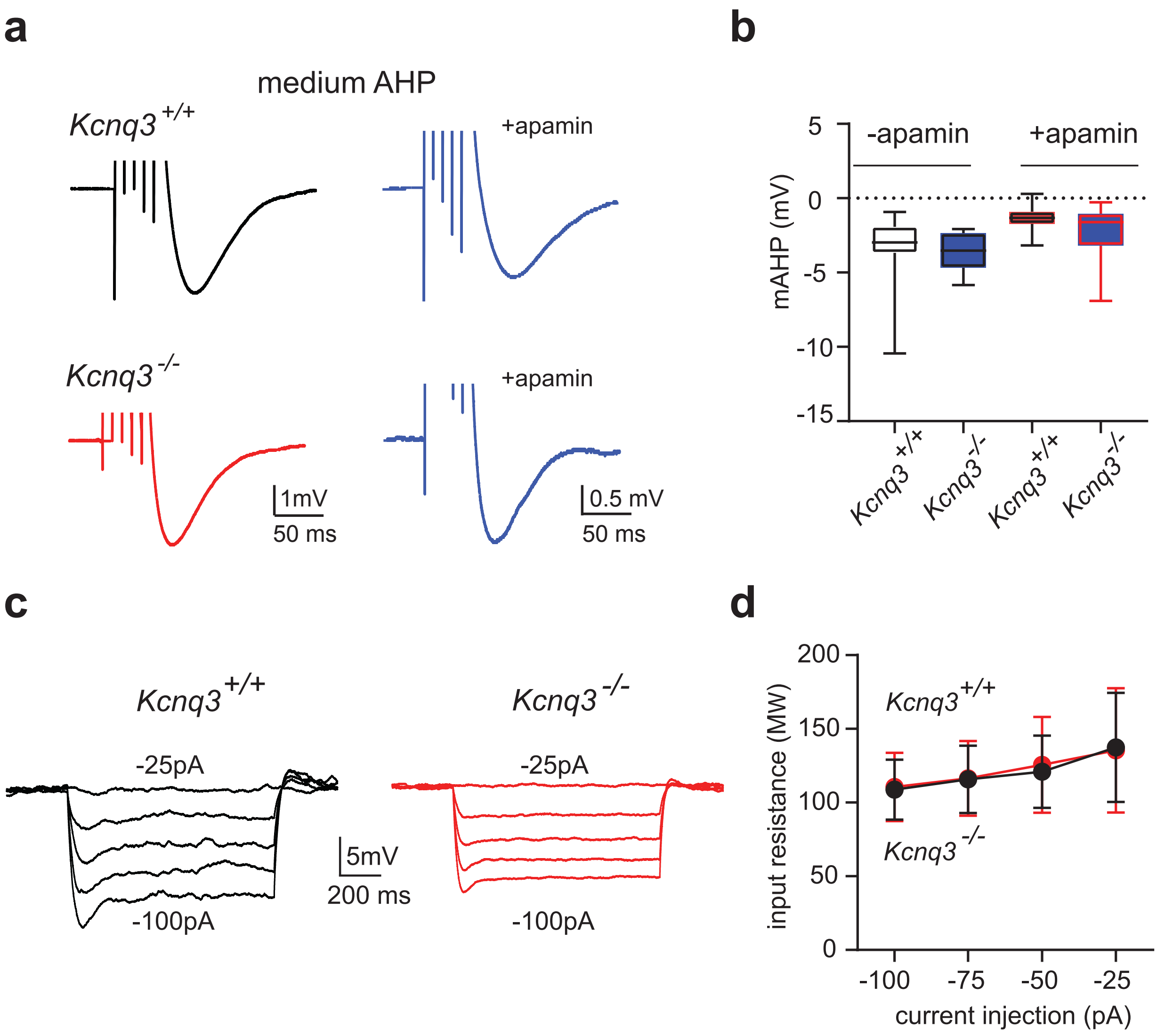

Supplementary Figure 2. Deletion of Kcnq3 does not impact the input resistance or medium afterhyperpolarization in CA1 pyramidal neurons. a Representatives traces showing the medium afterhyperpolarization (mAHP) following a brief train of current pulses (5 pulses 1 nA, 2 ms at 100 Hz) in either *Kcnq3*$^{+/+}$ or *Kcnq3*$^{-/-}$ CA1 pyramidal neurons before or after application of apamin (100 nM). **b** Summary data showing that *Kcnq3* ablation does not impair the mAHP (-apamin: *Kcnq3*$^{+/+}$, n=11 cells vs. *Kcnq3*$^{-/-}$, n = 10 cells, p = 0.49, Mann-Whitney-U-test; +apamin: *Kcnq3*$^{+/+}$, n=10 vs. *Kcnq3*$^{-/-}$, n = 9, p = 0.84). The center line in BW plots indicates the median, the top and bottom edges indicate the 25th and 75th percentiles, respectively, and the whiskers extend to the maximum and minimum data points. **c** Voltage responses to current injections (1 s pulse duration, -25 pA to -100 pA increments of 25 pA) from *Kcnq3*$^{+/+}$ (n=11) and *Kcnq3*$^{-/-}$ (n=12) CA1 pyramidal neurons. **d** Summary data showing that deletion of *Kcnq3* does not alter the input resistance of CA1 pyramidal neurons as measured in the soma (*Kcnq3*$^{+/+}$, n=11 cells vs. *Kcnq3*$^{-/-}$, n = 12 cells, p > 0.7, t-test). Data are presented as mean ± SEM.

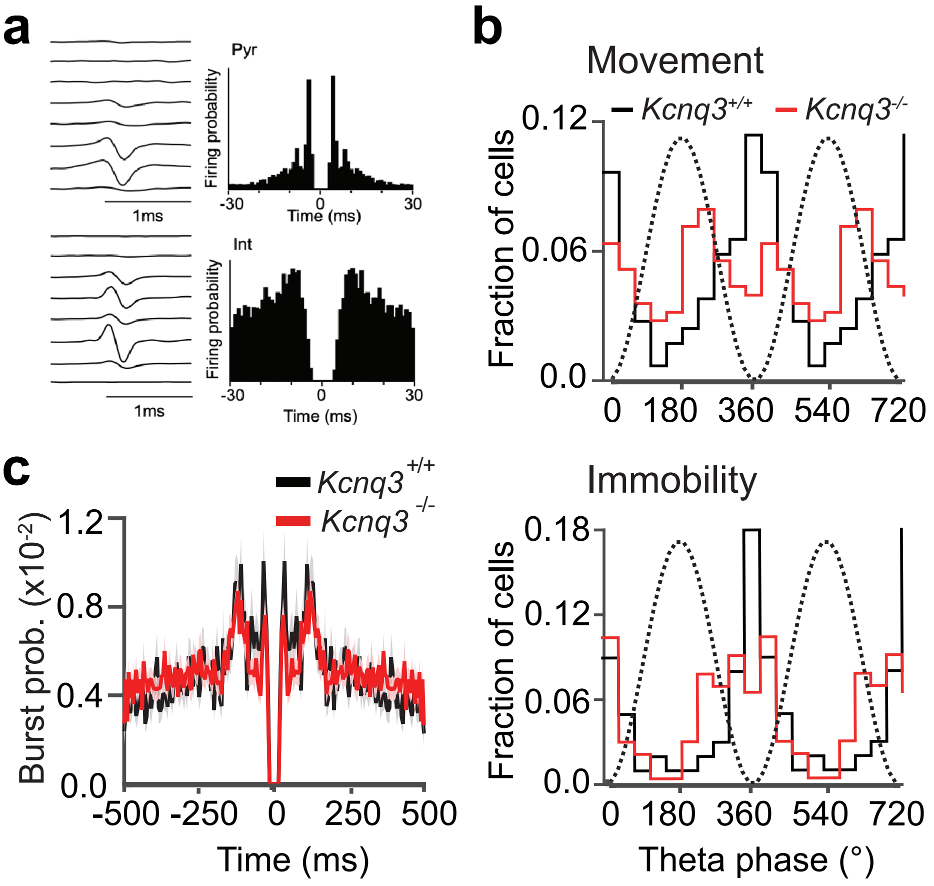

Supplementary Figure 3. Theta rhythmic coordination of neuronal discharge in constitutive *Kcnq3* knockout and wild-type mice. a Examples of isolated putative pyramidal cell (Pyr) and fast spiking interneuron (Int), single units. Average spike waveforms (left) recorded using a silicon probe and corresponding auto-correlograms (right). Bin width, 1 ms. Data are presented as mean ± SEM. **b** Histogram of preferred firing phases of putative pyramidal cells during spatial navigation (movement, *Kcnq3*$^{+/+}$, n = 145 cells; *Kcnq3*$^{-/-}$, n = 126 cells) and alert immobility (*Kcnq3*$^{+/+}$, n = 50 cells; *Kcnq3*$^{-/-}$, n = 115 cells), see Results for statistical comparisons. **c** Auto-correlation of burst times during theta oscillations (*Kcnq3*$^{+/+}$, n = 155 cells; *Kcnq3*$^{-/-}$, n = 279 cells, cumulative probability between 100 and 200 ms, p = 0.82, Mann-Whitney-U-test).

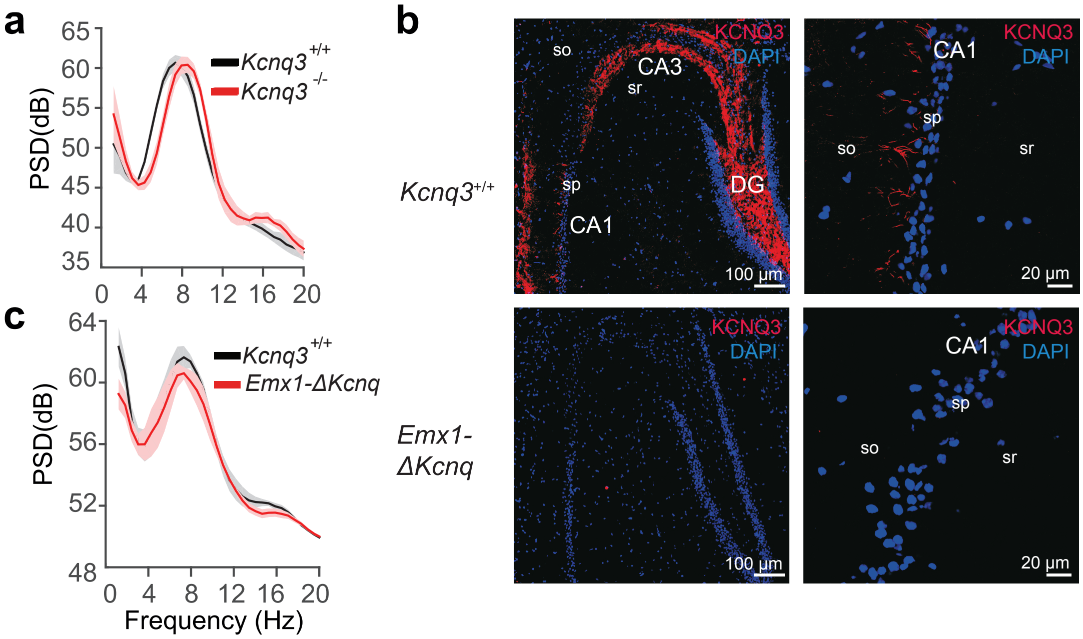

Supplementary Figure 4. Intact LFP theta oscillations in constitutive and conditional *Kcnq3* knockout mice. a Normalized power spectral density (PSD) of LFP during theta oscillations (*Kcnq3*$^{+/+}$, N = 5 mice, *Kcnq3*$^{-/-}$, N = 4 mice, cumulative theta band power, p = 0.63, t-test). **b** Kcnq3 immunofluorescence in hippocampus of wild-type and Emx1-Δ*Kcnq3* mice. S.p. – str. pyramidale, s.o. – str. oriens, s.r. – str. radiatum. **c** PSD of LFP during theta oscillations (Emx1-Δ*Kcnq3*, N=4 mice, *Kcnq3*$^{+/+}$, N=6 mice, cumulative theta power, p = 0.4, t-test). Data are presented as mean ± SEM.

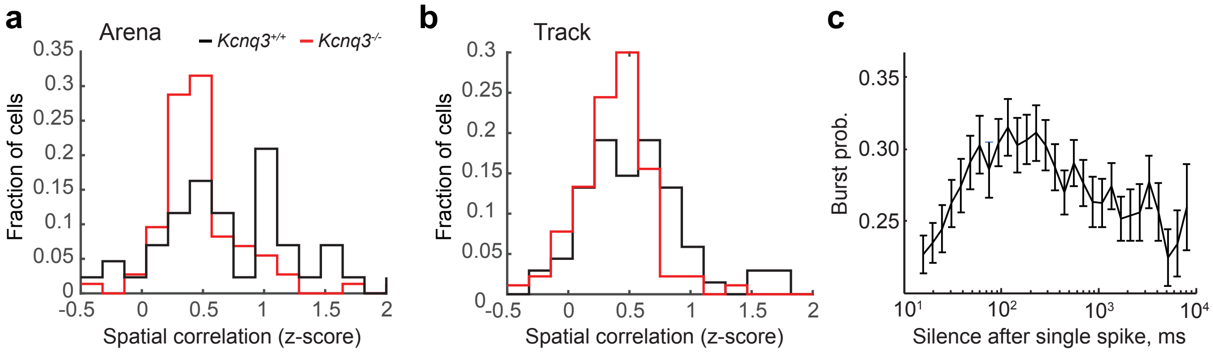

Supplementary Figure 5. a, b Distribution of spatial correlations of burst and single spike discharge in the arena (**a**) or on the circular track (**b**, z-transformed Pearson's correlation coefficients, arena: *Kcnq3*$^{+/+}$, n = 43 cells, *Kcnq3*$^{-/-}$, n = 73 cells; track: *Kcnq3*$^{+/+}$, n = 68 cells, *Kcnq3*$^{-/-}$, n = 90 cells), see Results for statistical comparisons. **c** Probability of burst emission as a function of silence duration after a single spike (*Kcnq3*$^{+/+}$, n = 81 cells). Data are presented as mean ± SEM.

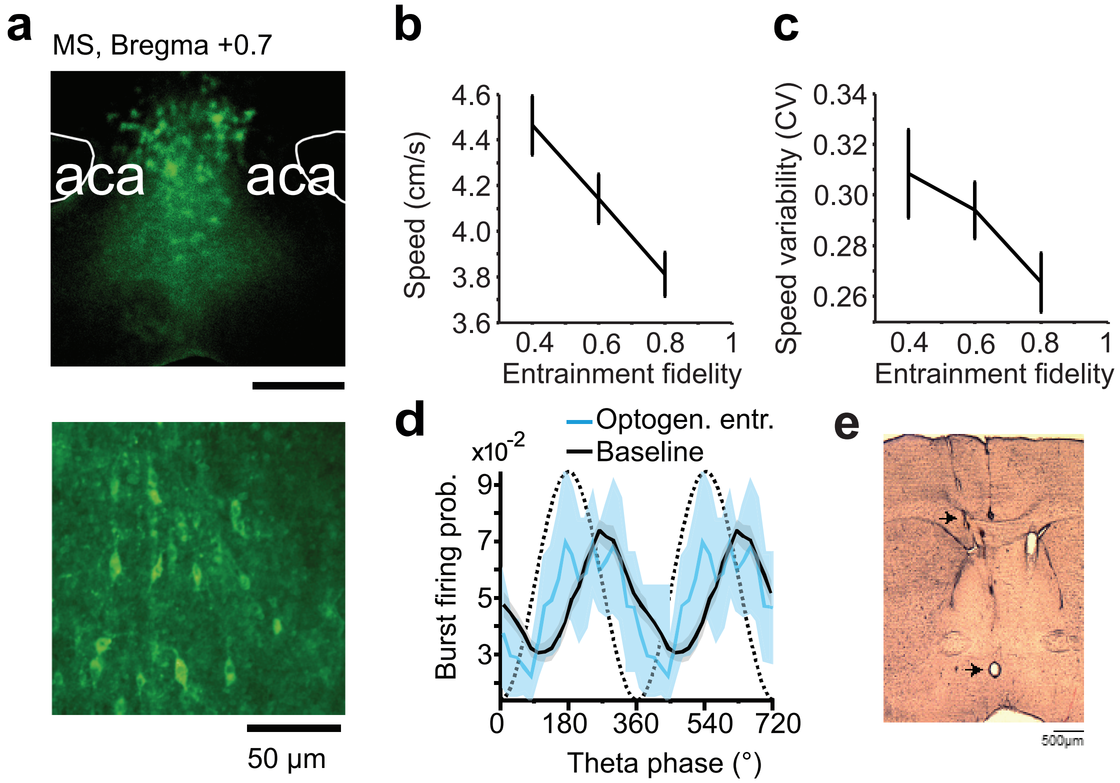

Supplementary Figure 6. **a** Expression of *AAV2/5.CAG.hChR2(H134R)-mCherry.WPRE.SV40* in the MS. **b,c** Representative examples of the association of the average running speed (**b**, Pearson's r = -0.22, p = 0.0012) and speed variability (**c**, r = -0.14, p = 0.04) with the fidelity of theta entrainment by optogenetic stimulation of $GABA^{+}$ and $GABA^{-}$ MS to hippocampus projections (n=213 stimulation epochs, 18 recording sessions in one mouse). **d** Burst probability during spontaneous theta oscillations and theta-entraining stimulation (n = 43 and 7 cells, respectively) according to the oscillation phase during phase-locked activation of $GABA^{+}$ and $GABA^{-}$ projections. ChR2 was expressed in neurons as well as in glial MS cells using *AAV.CAG.hChR2*. **e** A brain section showing a silicon probe track and a lesion in the MS (arrows). A part of the optic fibre track is visible in the contralateral hemisphere. Data are presented as mean ± SEM.

Supplementary Table 1

Oligonucleotide primers to detect mouse *Kcnq2* and *Kcnq3* transcripts in RT-PCR

Primer	Sequence
07396_kcnq2_fw	5´-AGGAAGCCGTTCTGTGTGA-3´
07397_kcnq2_rv	5´-GCAGAGGAAGCCAATGTACC-3´
07414_Q3KO_RT_fw	5´-CCTTTGCTATTTTCATCTTTGGAGC-3´
07415_Q3KO_RT_rv	5´-CCAGGCAGTGATGAGTTCTTTG-3´

Supplementary statistical analysis

For Fig. 1d Repeated two-way ANOVA with factors 'genotype' and 'injected current' revealed significant effects of the genotype ($F_{1,175}$ = 30.8, p < 0.0001, n = 11 cells from 4 *Kcnq3*$^{+/+}$ mice, n = 12 cells from 4 *Kcnq3*$^{-/-}$ mice) and of the injected current ($F_{7,175}$ = 52.5, p < 0.0001) on the number of evoked action potentials.

For Fig. 1e Repeated two-way ANOVA with factors 'genotype' and 'injected current' revealed significant effects of the genotype ($F_{1,164}$ = 48.6, p < 0.0001, n = 11 cells from 4 *Kcnq3*$^{+/+}$ mice, n = 12 cells from 4 *Kcnq3*$^{-/-}$ mice) and of the injected current ($F_{7,164}$ = 19.9, p < 0.0001) on the frequency of the last two action potentials in evoked trains. Also the frequency of the initial two action potentials was different between genotypes ($F_{1,166}$ = 7.2, p = 0.0080) and between amounts of the injected current ($F_{7,166}$ = 47.1, p < 0.0001) .

For Fig. 1g. Comparisons of the probability of bursts consisting of different number of spikes, depending on distributions normality using t-test or Mann-Whitney-U-test (Bonferroni adjusted α = 0.0083) revealed that the probability of 2-spike bursts was lower in pyramidal cells in *Kcnq3*$^{-/-}$ than in *Kcnq3*$^{+/+}$ (p < 0.0001) while the probabilities of longer bursts, i.e. consisting of 3, 4, 5, 6 or 7 spikes, were higher in the mutant than in controls (p < 0.0001). n = 299 cells from 6 *Kcnq3*$^{+/+}$ mice; n = 413 cells from 4 *Kcnq3*$^{-/-}$ mice.

For Fig. 1h. The first order spike accommodation rate was defined as $\mathrm{R_{accom}} = (2_{\mathrm{nd}}\mathrm{ISI} - 1_{\mathrm{st}}\mathrm{ISI})/1_{\mathrm{st}}\mathrm{ISI}$, where $1_{\mathrm{st}}\mathrm{ISI}$ and $2_{\mathrm{nd}}\mathrm{ISI}$ are the first and the second inter-spike intervals within the same burst, respectively. Repeated two-way ANOVA with factors 'genotype' and 'burst length' revealed a significant interaction between the factors ($F_{2,44766}$ = 6.2, p = 0.0021) as well as an effect of genotype on the first order spike accommodation ($F_{1, 44766}$ = 90.0, p < 0.0001). n = 305 cells from 6 *Kcnq3*$^{+/+}$ mice; n = 413 cells from 4 *Kcnq3*$^{-/-}$ mice.

For Fig. 1i. Comparison of the average ISI in a burst using two-way ANOVA with factors "genotype" and "burst length" revealed a significant interaction between the factors ($F_{4,7604}$ = 18.8, p < 0.0001) as well as an effect of genotype on the average ISI (*Kcnq3*$^{+/+}$, 7.42 ± 0.04 ms, n = 305 cells, N = 6 mice, *Kcnq3*$^{-/-}$, 6.67 ± 0.02 ms, n = 413 cells, N = 4 mice, $F_{1,7604}$ = 195.2, p < 0.0001). Pairwise t-tests or Mann-Whitney-U-tests of ISI between bursts consisting of 2, 3, 4, 5 and 6 spikes showed no significant difference in controls (Bonferroni adjusted α = 0.005, 0.0119 ≤ p ≤ 0.78). In *Kcnq3*$^{-/-}$ cells, average ISI differed between bursts of progressively increasing length (2- vs. 3-, 3- vs. 4-spikes etc., p < 0.0001, except for 5- vs. 6-spike bursts: p = 0.86).

For Fig. 2b,c. Power spectrograms (Fig. 2c) were computed for average cross-correlations (CCGs) of burst times (Fig. 2b) and for surrogate averaged CCGs in each genotype. Surrogate

CCGs were generated using a predictor shift (trial shuffling) method, by randomly permuting blocks of time stamps between the detected theta epochs 10000 times. Average power spectrum in -200 to 200 ms interval was computed for each spectrogram. The magnitude of the theta-band peak was significantly higher than spurious power peaks in *Kcnq3*$^{+/+}$ (SD above mean, 2.46, Gaussian percentile, p = 0.007, n = 154 cell pairs from 6 mice) but not in *Kcnq3*$^{-/-}$ (0.71, p = 0.24, n = 854 cell pairs from 4 mice, 99% CI = [-0.63 0.98] in 100 random subsamples n = 154 pairs each).

For Fig. 2f. Squared differences of firing probability computed in each theta phase bin between *Kcnq3*$^{-/-}$ and *Kcnq3*$^{+/+}$ populations of pyramidal cells during the course of one theta cycle were significantly higher for movement- than for immobility-associated theta oscillations (p=0.0049, t-test). *Kcnq3*$^{+/+}$, movement: n=191 cells, immobility: n=124 cells; N = 6 mice, *Kcnq3*$^{-/-}$, movement: n=209 cells, immobility: n=210 cells, N = 4 mice.

For Fig. 3b. Comparisons of the place field size between genotypes revealed no significant difference of place fields fired by all spikes (arena: $F_{1,167}$ = 0.12, p = 0.73, ANOVA, n = 76 cells from 5 *Kcnq3*$^{+/+}$ mice, n = 95 cells from 4 *Kcnq3*$^{-/-}$ mice; circular track: $F_{1,168}$ = 3.14, p = 0.067, ANOVA, *Kcnq3*$^{+/+}$, n = 78 cells, *Kcnq3*$^{-/-}$, n = 94 cells). Place fields generated by spikes fired in bursts tended to be larger in the mutant than in the control in the arena and were significantly increased in size on the track (arena, p = 0.082, track, p = 0.013, Mann-Whitney-U-Test). The size of place fields signaled by single spikes was reduced in both enclosures in *Kcnq3*$^{-/-}$ (arena, p = 0.0004, track, p < 0.0001, Mann-Whitney-U-Test).

For Fig. 3c,d. Delays of first single spikes fired after each burst were represented for each cell in a histogram (lags: 15 ms to 4 s). The histogram was normalized by the total number of single spikes (n = 117 cells from 6 *Kcnq3*$^{+/+}$ mice; n = 167 cells from 4 *Kcnq3*$^{-/-}$ mice).

For Fig. 4h. Squared differences of firing probability computed in each theta phase bin between populations of pyramidal cells recorded during spontaneous and optogenetically entrained theta oscillatory epochs were significantly higher in $GABA^+$ than in combined $GABA^+$ and $GABA^-$ projection stimulation (p = 0.0055, t-test). $GABA^+$, spontaneous theta, n=14 cells, optogenetically entrained theta, n=18 cells, N = 3 mice. $GABA^+$ and $GABA^-$, spontaneous theta, n=37 cells, optogenetically entrained theta, n=38 cells, N = 3 mice.

For Fig. 4l. MS theta phases of spikes were computed during local theta oscillations in baseline for putative $ChAT^+$ and $ChAT^-$ MS cells. Phase histograms were computed for each cell and normalized by subtraction of its mean. Normalized histograms were concatenated 10000 times, power spectra were calculated (window size=512, NW=3, sampling rate = [nbins in a cycle] x 7.5 Hz (5-10 Hz bandwidth)) and normalized by the cumulative power in the 0-20 Hz band. Theta phase modulation index was defined as the maximum power in theta band.
