# Supplementary material for: Place fields of single spikes in hippocampus involve Kcnq3 channel-dependent entrainment of complex spike bursts
Source: Nat Commun. 2021 Aug 10;12:4801. doi: 10.1038/s41467-021-24805-2 (PMC8355348; doi:10.1038/s41467-021-24805-2)
Supplement: Supplementary file 3 — Reporting Summary [file 41467_2021_24805_MOESM3_ESM.pdf]

nature portfolio

Corresponding author(s): Thomas J. Jentsch, Alexey Ponomarenko

Last updated by author(s): 06/14/2021

Reporting Summary

Nature Portfolio wishes to improve the reproducibility of the work that we publish. This form provides structure for consistency and transparency in reporting. For further information on Nature Portfolio policies, see our Editorial Policies and the Editorial Policy Checklist.

Statistics

For all statistical analyses, confirm that the following items are present in the figure legend, table legend, main text, or Methods section.

n/a	Confirmed	
☐	☒	The exact sample size (n) for each experimental group/condition, given as a discrete number and unit of measurement
☐	☒	A statement on whether measurements were taken from distinct samples or whether the same sample was measured repeatedly
☐	☒	The statistical test(s) used AND whether they are one- or two-sided<br>*Only common tests should be described solely by name; describe more complex techniques in the Methods section.*
☐	☒	A description of all covariates tested
☐	☒	A description of any assumptions or corrections, such as tests of normality and adjustment for multiple comparisons
☐	☒	A full description of the statistical parameters including central tendency (e.g. means) or other basic estimates (e.g. regression coefficient) AND variation (e.g. standard deviation) or associated estimates of uncertainty (e.g. confidence intervals)
☐	☒	For null hypothesis testing, the test statistic (e.g. F, t, r) with confidence intervals, effect sizes, degrees of freedom and P value noted<br>*Give P values as exact values whenever suitable.*
☒	☐	For Bayesian analysis, information on the choice of priors and Markov chain Monte Carlo settings
☒	☐	For hierarchical and complex designs, identification of the appropriate level for tests and full reporting of outcomes
☐	☒	Estimates of effect sizes (e.g. Cohen's d, Pearson's r), indicating how they were calculated

Our web collection on statistics for biologists contains articles on many of the points above.

Software and code

Policy information about availability of computer code

Data collection	Cheetah (Neuralynx).
Data analysis	Mathworks MATLAB, 2010, 2014, custom code; NeuroSuite (NDManager, Klustakwik, Klustres), GraphPad Prism 8; MySQL 5.7; MS Excel 16, Molecular Devices Clampfit 10.7, Zeiss ZEN 2009, Adobe Photoshop CS6.

For manuscripts utilizing custom algorithms or software that are central to the research but not yet described in published literature, software must be made available to editors and reviewers. We strongly encourage code deposition in a community repository (e.g. GitHub). See the Nature Portfolio guidelines for submitting code & software for further information.

Data

Policy information about availability of data

All manuscripts must include a data availability statement. This statement should provide the following information, where applicable:

- Accession codes, unique identifiers, or web links for publicly available datasets
- A description of any restrictions on data availability
- For clinical datasets or third party data, please ensure that the statement adheres to our policy

Source data underlying Fig. 1-4, Tables 1,2 and Suppl. Fig. 1-6 are provided with the paper (Source Data file). Spike trains recorded in Kcnq3-/- and Kcnq3+/+ mice were made available via Figshare (DOI).

Field-specific reporting

Please select the one below that is the best fit for your research. If you are not sure, read the appropriate sections before making your selection.

☒ Life sciences ☐ Behavioural & social sciences ☐ Ecological, evolutionary & environmental sciences

For a reference copy of the document with all sections, see nature.com/documents/nr-reporting-summary-flat.pdf

Life sciences study design

All studies must disclose on these points even when the disclosure is negative.

Sample size	Sample size was based on previous studies from our and other labs (Wulff et al., PNAS, 2009, Carus-Cadavieco et al., Nature, 2017, Mederos et al., Nature Neurosci., 2021). No statistical tests were used to predetermine sample sizes.
Data exclusions	Putative pyramidal cells: multiunits (without a clear refractory period of <3 ms) were excluded from analyses (Nádasdy et al., J Neurosci., 1998, Harris et al., J Neurophysiol., 2000). Except for this no data were excluded from analyses.
Replication	All results reported in this study are based on recordings of multiple independently recorded cells. All attempts at replication were successful.
Randomization	Animals were pseudorandomly assigned to experimental and control groups before surgery and experiments.
Blinding	The experimenter was blind to the genotypes of mice during all experiments, except for in vivo recordings during the revision when collecting data related to specific requests could not be done blind. Data preprocessing (semi-automatic spike sorting) was always performed by genotype-blind researchers.

Reporting for specific materials, systems and methods

We require information from authors about some types of materials, experimental systems and methods used in many studies. Here, indicate whether each material, system or method listed is relevant to your study. If you are not sure if a list item applies to your research, read the appropriate section before selecting a response.

Materials & experimental systems

n/a	Involved in the study	
☐	☒	Antibodies
☒	☐	Eukaryotic cell lines
☒	☐	Palaeontology and archaeology
☐	☒	Animals and other organisms
☒	☐	Human research participants
☒	☐	Clinical data
☒	☐	Dual use research of concern

Methods

n/a	Involved in the study	
☒	☐	ChIP-seq
☒	☐	Flow cytometry
☒	☐	MRI-based neuroimaging

Antibodies

Antibodies used	The following primary KCNQ3 antibody was used: rabbit anti-KCNQ3 (named Q3Crb129, 1:200). The secondary goat anti-rabbit Alexa 555 antibody (1:1000) was from Invitrogen.
Validation	Tzingounis, A.V. et al. The KCNQ5 potassium channel mediates a component of the afterhyperpolarization current in mouse hippocampus. Proceedings of the National Academy of Sciences 107, 10232-10237 (2010).

Animals and other organisms

Policy information about studies involving animals; ARRIVE guidelines recommended for reporting animal research

Laboratory animals	Twenty-seven C57Bl/6J, Kcnq3-/-, Emx1 cre/cre male mice, older than 12 weeks were used.
Wild animals	No wild animals were used in the study.
Field-collected samples	No wild animals were used in the study.
Ethics oversight	Landesamt für Gesundheit und Soziales (LaGeSo), Berlin, Germany, Regierung von Unterfranken, Würzburg, Germany

Note that full information on the approval of the study protocol must also be provided in the manuscript.
